# Proposed modified Brandwein-Gensler histological risk assessment score as prognosticator in oral squamous cell carcinoma

**DOI:** 10.4317/medoral.27743

**Published:** 2025-11-22

**Authors:** Deepti Sharma, Shruti Gupta, George Koshy, Kanwardeep Singh Kwatra, Vishal Kumar Sharma, Atampreet Kaur

**Affiliations:** 1 Oral Pathology and Microbiology, Christian Dental College &amp; Hospital, Ludhiana, Punjab; 2 Department of Oral Anatomy, Post Graduate Institute of Dental Sciences, Rohtak, Haryana, India; 3 Department Labs, Department of Pathology, Mohan Dai Oswal Cancer Hospital, Ludhiana; 4 Department of Orthodontics, Desh Bhagat Dental College and Hospital, Mandi Gobindgarh, Punjab, India

## Abstract

**Background:**

Histopathological parameters significantly impact the prognosis and treatment strategies of oral squamous cell carcinoma (OSCC). Various risk assessment scores and grading systems have been proposed in the past, primarily based on subjective assessment of microscopic features. To modify Brandwein-Gensler risk score by including the worst pattern of invasion-5, perineural invasion and percentage stromal tumor infiltrating lymphocytes(TILs) and evaluate the relationship of the modified score with clinicopathological variables.

**Material and Methods:**

This retrospective observational study was conducted on 58 OSCC patients. The density of stromal TILs was determined on routine microscopic sections using image analysis software, thereby quantifying lymphocytic host response as percentage stromal lymphocytes. Furthermore, the modified Brandwein-Gensler risk score was derived from the assessment of the worst pattern of invasion-5, perineural invasion and percentage stromal tumor infiltrating lymphocytes. Chi-square test and Univariate analysis were used to find the association of categorical and continuous variables. The Kaplan-Meier and log-rank tests were used to estimate the survival rates.

**Results:**

A significant positive association of modified Brandwein-Gensler score was observed with pathological node (p=0.005), tumor grading (p=0.025), depth of invasion(p=0.016) and overall survival (p=0.03), signifying that high score tumors showed poor differentiation, invasive features and lower survival time.

**Conclusions:**

A modified, simplified and objective version of Brandwein Gensler histological risk assessment score is derived, revealing a significant association with tumor differentiation, depth of invasion and lymph node involvement.

## Introduction

Precision medicine warrants appraising characteristics of both the tumor and surrounding stromal microenvironment using multiple parameters for accurate diagnostic and prognostic assessment (1). Management of oral squamous cell carcinoma (OSCC) patients relies on the tumor, node and metastasis (TNM) staging system, which reflects heterogeneous prognosis; consequently, many patients (12-14%) experience relapses and have a high morbidity and mortality due to locoregional or distant recurrences (2). This highlights the necessity to explore histopathological or molecular predictive and prognostic markers capable of effectively stratifying disease biology (3). Recent research into OSCC underscores the prognostic importance of various histopathological parameters, including depth of invasion (DOI), perineural invasion (PNI), lymphovascular invasion (LVI), tumor histological grade, worst pattern of invasion (WPOI), lymphocytic host response (LHR), mitotic activity/index (MI), tumor thickness, tumor budding, tumor-stroma ratio and tumor infiltrating lymphocytes (TILs), all of which significantly influence clinical decision-making and risk assessment (4 , 5). The expedition for reliable, rapid, reproducible and straightforward markers of immune response highlights TILs as valuable immune biomarker offering meaningful insights for refining treatment strategies (6). Several histological scoring systems have been standardized and established in the past to estimate disease burden in OSCC. The Brandwein Gensler (BG) risk model, propose in 2005 as a modification and extension of previous histological models, includes 3 main factors i.e WPOI, LHR, and PNI for risk categorization, providing a more comprehensive assessment of tumor and its microenvironment (7 , 8). However, it requires detailed analysis and cumulative scoring of 3 parameters with further sub categorization, which might be time consuming in routine practice, and also there is a subjective estimation of immune infiltrate (9 , 10). The density of TILs can be assessed on microscopic sections without evaluating the subsets of lymphocytes by the new methods proposed by the International Immuno-Oncology Biomarkers Working Group, thus facilitating the quantification and objective estimation of the immune response (11). In addition, both BG score and TIL evaluation can be applied to the routine histopathological examination without any additional financial burden or time constraints, and thus serve as alternatives for complex and costly molecular markers and genetic signatures (12 , 10 , 9). In addition, WPOI-5 and PNI have been considered as independent prognosticators in OSCC (13 , 7 , 8). Multiple studies have evaluated the BG score and TILs independently, correlated them with clinicopathological characteristics and survival outcomes, with varied findings and shortcomings. But none of the studies have attempted a combined evaluation, so this study intends to address this research gap (14 , 11 , 7 , 6). Therefore, the present novel pioneering study was conducted with a rationale to simplify, modify and objectify the BG score by including quantitative evaluation of TILs as an independent variable, substituting the LHR, PNI, and WPOI-5 only. Thus, it has been hypothesized that substituting the pattern of lymphocytic infiltrate with percentage (%) stromal TILs density as a continuous and quantitative parameter may provide more biologically relevant information, objective results with minimal interobserver variability, and allow more accurate statistical assessment. Thus, the objectives of this study were to quantify LHR by determining the %stromal TILs and modify the BG score by including %stromal TILs, PNI and WPOI-5 as independent histopathological parameters. In addition, the relationship of the aforementioned modified score and %stromal TILs with clinicopathological variables and overall survival (OS) was also assessed.

## Material and Methods

The present retrospective observational study was conducted in the Department of Oral and Maxillofacial Pathology, Christian Dental College, Ludhiana, in full accordance with ethical principles and with approval of the Institutional Review Board. The study protocol was approved by the Institutional Research Ethics Committee (IECBMHR/202411-535/Apprvl-B-Gesler/CDC).

The patients' medical data were anonymized prior to access and analysis.

Sample size: From the given prevalence of oral cancer by GLOBCAN 2018 (15) Indian data, the size of the sample was calculated using the formula: n=Z /2 2 *P(1-P)/d2, where Z /2 is the critical value of the normal distribution at /2, P is prevalence and d is margin of error. For the present research, taking into consideration the confidence level of 95%, of 0.05, the critical value of 1.96, the prevalence of 10.4% and the margin of error of 7%, the minimum sample size came out to be 54.

Selection of Participants

58 histopathologically confirmed OSCC patients without any prior treatment and with tumor located in the oral cavity (tongue, gingiva, buccal mucosa, alveolus, floor of the mouth or hard palate) were included in the study. Clinical details [age, gender, site, lymph node status, staging (AJCC 8th edition staging system)(16), histological grading (WHO)(16) and survival data were gathered from medical records of the patients. Patients with insufficient clinical data and with any previous treatment (chemotherapy/radiotherapy) for the present malignancy were excluded.

Light microscopy

Formalin-fixed, paraffin-embedded blocks and hematoxylin and eosin (H&amp;E) stained slides from the resected specimens of all patients included in the study were retrieved from the departmental archives. Differentiation status of all the cases was assessed, and slides were evaluated for WPOI-5, PNI and density of TILs. The criteria for determining WPOI-5 are the presence of secondary tumor nodules, measuring at least 1mm apart from the primary tumor (under 20x magnification) or the nearest adjacent satellite (17). PNI is characterized by the presence of tumor cells around one-third of the nerve or the presence of tumor cells inside the epineurium, perineural space, or nerve sheath (18).

TIL evaluation

**Figure 1:**
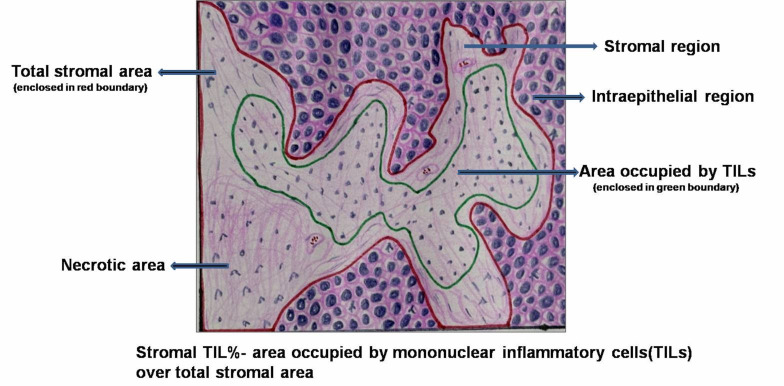
Diagrammatic representation of TIL assessment.

**Figure 2:**
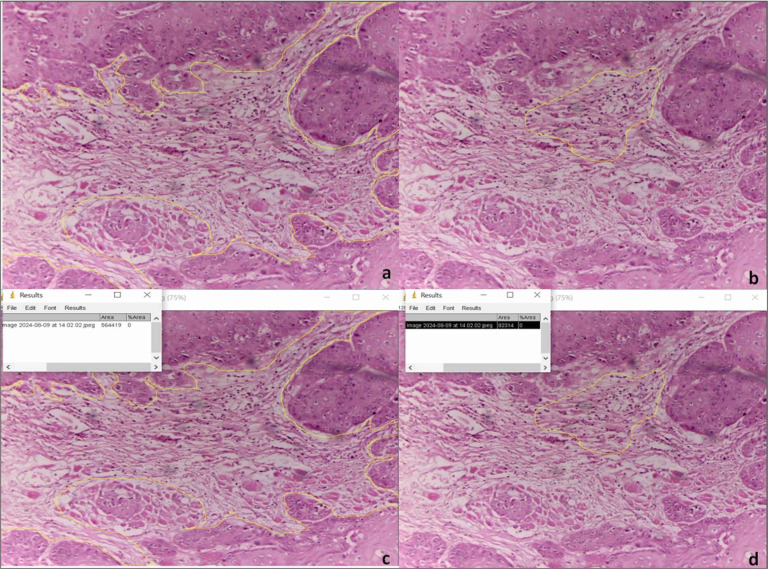
Micropictographs (a-d) depicting markings of stromal and TIL areas.

The TILs density was determined on H&amp;E-stained sections based on the recommendation by the International Immuno-Oncology Biomarkers Working Group (19). The slides were first scanned at low magnification (4x); thereafter, images were acquired with a CCD camera using a 20× objective and transferred to a computer. Digitalized images of 5 representative fields were used to calculate the percentage TIL stromal area using image analysis software (Image J) (20). Total stromal area and area occupied by TILs were marked manually.(Figure 1 and Figure 2) All of the mononuclear cells, including the lymphocytes in the stromal area, were defined as TILs. However, areas occupied by granulocytes and other polymorphonuclear leukocyte, ulcerated, crushed or necrotic areas were excluded. The %stromal TILs area was calculated by the formula: Fraction of the area occupied by mononuclear inflammatory cells/ the total stromal area *100. The average value of 5 fields was considered as the final score for the slide. So, %stromal TILs represent the proportion of the tumor's stroma that is occupied by lymphocytes.

1

2

Determination of Modified BG score

Modified BG score was derived by the assessment of the WPOI-5 as present/absent (score1/0), PNI present/absent (score1/0) and TIL density as %stromal TILs low/high (1/0). Median TIL value of all cases was used as a cut-off to divide the patients into high and low risk categories. To derive the modified BG score, the scores of all 3 parameters were added.

Statistical analysis

**Figure 3:**
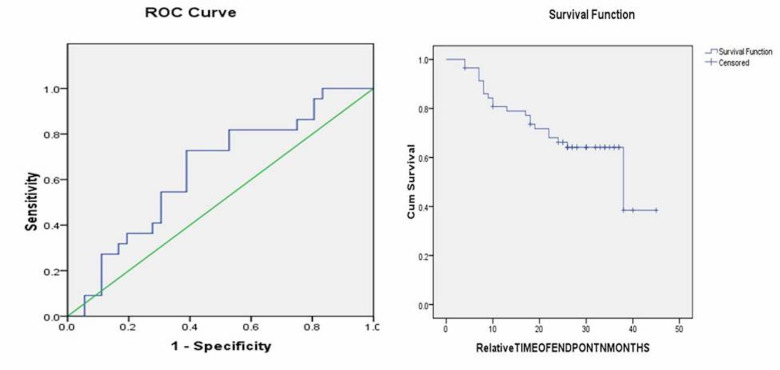
Receiver operating characteristics (ROC) curve and Survival function for % stromal TILs.

**Figure 4:**
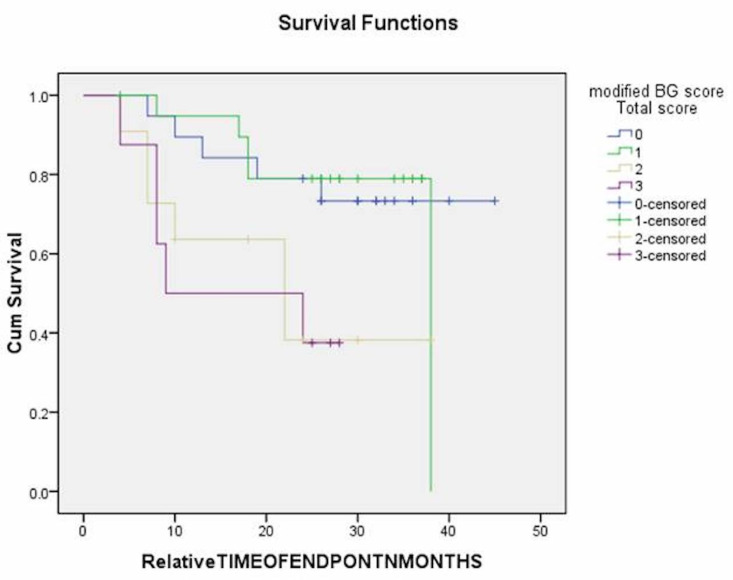
Kaplan Meier survival estimates for overall survival according to the modified BG score in oral cancer.

All statistical analysis was performed using SPSS (Statistical Packages for Social Sciences, version 21.0. Armonk, NY: IBM corp.) Categorical data were represented as count (percentage) and continuous data as mean±SD or median. Pearson chi-square test was used to find the association of categorical variables, and the univariate analysis was used to find the association of continuous variables in more than 2 groups. Pearson correlation coefficient was applied to find the correlation between the variables. Receiver operating characteristics (ROC) analysis (Figure 3) was used to estimate the predictive value of %stromal TILs. The Kaplan-Meier and log-rank tests were used to estimate the survival rates (Figure 4). The p-value &lt;0.05 was considered significant.

3

4

## Results

Patient and tumor characteristics

A total of 58 patients were included in the study, of which 48 (82.8%) were males and 10 (17.2%) females. The mean age of the patients in the selected cohort was 52 years, with an age range of 28-76 years. 50% of the patients had carcinoma of the lateral border of the tongue. Table 1. provides the detailed patient and disease characteristics.

Table 1

Interpretation regarding %stromal TILs

In the present study, the mean and median value of %stromal TILs was 22.8±12.7 and 18. Based on the median value of TILs patients were further categorized into low (TILs18) and high (TILs&gt;18) TIL groups. Both groups had an equal distribution of subjects, with 29 subjects in each group. A statistically significant difference was observed in mean %stromal TILs with regard to different age groups (p=0.001); however, no significant findings were observed with all other studied parameters. (Table 2) In addition, no significant correlation was found between % age stromal TILs and DOI (p=0.20).

Table 2

Modified BG score evaluation and interpretation

Modified BG score was derived for all the cases, and a significant positive association of modified BG score was observed with pN(pathological node) (p=0.005), tumor grading (p=0.025) and DOI (p=0.016), however, this association was not significant for pStaging, cStaging (clinical staging) and cN (clinical node). In addition, no significant relation was seen between modified BG score and demographic variables (age, gender and site). (Table 3.)

Based on the modified BG score, all the cases were categorized into high (score 2-3) and low risk (score 0-1) categories, comprising 19 and 39 patients, respectively. A significant difference was observed in different modified BG risk score categories concerning pN (p=0.02), cStaging (p=0.02), cN (p=0.01), grading (p=0.04) and DOI (p=0.02). (Table 3)

Table 3

DOI findings

In this study the mean value of DOI was observed to be 1.13±0.67. A significant difference was observed in mean DOI regarding different categories of site (p=0.000), pN (p=0.003), pStaging (p=0.04), and modified BG scores (p=0.02). (Table 2)

Survival analysis

ROC analysis and area under the curve (AUC) revealed that %stromal TILs was not a significant predictor of survival. (Figure 2) Kaplan-Meier analysis and log-rank pairwise comparison revealed a significant association of modified BG score with OS (p=0.03). (Figure 4) Mean survival estimates were found to be 37,33,23, and 17 months corresponding to modified BG scores 0,1,2 and 3, respectively, showing low-score patients lived for longer duration.

## Discussion

Recent evidence has indicated a paradigm shift towards tumor microenvironment (TME), host tumor interface and invasive histopathological features as key prognosticators, necessitating a more holistic approach to tumor assessment (21). It has been observed that the same stage tumors might behave differently, and low-stage cancer might be enigmatically worse despite complete removal, ensuing invasive histology (22 , 23). Tumors have been categorized as immunologically "cold" or "immune desert" (lacking immune cells), "hot" or "inflamed" (tumor with diffuse distribution of immune cells) and "excluded" (immune cells at tumor boundaries). These immunophenotypes of tumor definitely impact the survival, and TILs are considered markers of immune response (24). Therefore, scientific work focusing on TILs as another potential biomarker in many solid malignancies, as well as OSCC, is expanding (25). Also, research is focused on developing predictive models encompassing multiple parameters to determine the disease outcome based purely on histological evaluation (8).

So, a comprehensive risk assessment in OSCC could help to stratify high/low risk patients, determine the possible consequences and likelihood of poor outcome, which further facilitates planning appropriate therapeutic strategies to effectively reduce disability and death rates (2). A recently conducted meta-analysis and other published data have substantiated pTNM (pathological TNM), tumor volume, medullary bone invasion, LVI, surgical margin status, regional lymph node and distant metastasis, PNI, DOI, extranodal extension, tumor thickness, tumor budding, tumor-stroma ratio and pattern of invasion as definite, easily assessable, and unswerving key prognosticators in OSCC (26 , 27). So far, the majority of the risk scoring criteria have included qualitative assessment of LHR, but now the inclination is towards quantitative evaluation (22 , 28 , 29). Xu et al. (2021) favored the higher prognostic potential of stromal TILs, a quantitative histologic marker of tumoral immune response over the qualitative estimation of LHR. LHR is a pre-determined three-tiered qualitative system evaluating the lymphocytic response of tumor front, whereas TILs, as a quantitative variable, facilitate determining the ideal cut-off using ROC analysis and evaluating the entire tumor area, thus precise appraisal of the host immune response (10). Therefore, the present study contemplated %stromal TILs as one of the quantitative parameters replacing the LHR in BG risk assessment score.

The present study assessed the TIL density as %stromal TILs using the standardized methodology given by the International Immuno-Oncology Biomarkers Working Group (30 , 11). Although the association between %stromal TILs and most clinicopathological variables was found to be statistically non-significant but a trend of high %stromal TILs in low-grade cases and low %stromal TILs in association with lymph node metastasis was observed. In addition, %stromal TILs was not found to be a significant predictor of survival, and showed no significant association with any clinical or histological parameters except age. The findings were in concordance with Ahuja et al. (31) who also observed no significant association of TILs density with age, gender, site, grade, nodal status and PNI; however, they found a significant association with tumor size and LVI. In contrast to our findings, other studies found a significant relation of TILs with OS, disease-free survival (DFS) and disease-specific survival (DSS) in head and neck cancer and OSCC (19 , 32 , 33). The discrepancy in available literature could be due to non-assessment of intratumoral and invasive front TIL density (varied regions of evaluation), different software used for TIL assessment, differences in the number of cases and variation in cut-off criteria. Additionally, the restricted availability of literature about TIL assessment using H&amp;E-stained sections in OSCC forbade the use of established cut-off values. Even though DOI showed no significant relation with %stromal TILs, a significant association was seen with pTNM and pN, pointing out that high stage tumors with aggressive microscopic features and nodal involvement indicate aggressive tumor biology in agreement with the literature (34).

BG histological risk assessment score included WPOI and PNI as key parameters with further subcategorizations (35). But current evidence suggests WPOI-5 and PNI (present or absent) as a significant independent prognostic factor for DFS and local tumor control in OSCC (36). The literature reported a significant association between survival (OS and DSS) and WPOI-5 (37). In addition, WPOI-5 has been associated with a higher risk of lymph node metastasis (36). Therefore, this study considered WPOI-5 only and PNI as independent variables. Notably, a significant relation of TILs has also been observed with WPOI and PNI. (6) Hence, the present study modifies the BG score and proposes a two-tier scoring system, which includes %stromal TILs: High or low, WPOI-5 and PNI as present or absent.

A significant association of modified BG score was observed with tumor grading, DOI and OS, addressing the fact that high score tumors showed poor differentiation, invasive features and lower survival time. Low and high risk modified BG score categories also revealed a significant association with DOI, clinical staging and clinical/pathological nodal involvement and degree of differentiation. High-risk patients are more likely to have nodal involvement, poor tumor differentiation and aggressive tumor biology. Discerningly, a significant relation was observed with many of the studied parameters, with the inclusion of %stromal TILs as one of the parameters of the modified BG score, along with PNI and WPOI-5.

This is pioneering research to simplify and objectify the histological scoring criteria and proposes a simple and cost-effective criterion, which could be routinely used and act as an adjunct to facilitate patient stratification and treatment selection. The strength of the present study is the selection of excisional biopsy specimens only which eliminates sample bias and allows thorough microscopic evaluation of the tumor.

The major limitations of the present study were its retrospective nature, site heterogeneity and small sample size. The information regarding possible confounding factors, like tobacco habits and alcohol, is lacking. Additionally, this study used only, H&amp;E staining as the method of pathological analysis, which limits determining the phenotypic variation and spatial distribution of lymphocytes, so findings could be deemed exploratory. In addition, the study design did not include other histological variables which could impact survival. Survival information was obtained from the records only and long-term follow-up was not done. Thus, for future analysis, prospective clinical trials with a larger cohort size, long-term follow-up and evaluation of TILs in 3 zones (stromal, intraepithelial and invasive front) and assessment of inter-rater concordance are recommended. To elucidate the true utility of TILs in guiding treatment decisions, more data should be extracted and evaluated pertaining to TIL evaluation with other diagnostic approaches. So, a collective evaluation of clinical staging and tumor histology would precisely predict the disease outcome and guide precision medicine, benefiting the patients.

## Conclusions

This study was an attempt to derive a modified, simplified and objective version of the BG histological risk assessment score, which could facilitate patient stratification and theragnostic care. Modified scores showed a significant association with tumor differentiation, DOI and lymph node involvement. Notably, the assessment of TILs was deemed feasible with just H&amp;E staining, emphasizing the importance of standardizing and establishing a simple evaluation method. Thus, this study warrants the external validation of its findings with multicentric approach.

## Figures and Tables

**Table 1:** Table Patient and disease characteristics of the studied cohort.

Serial No.	Parameters		Number of Subjects
1	Age	≤50 years	27
		>50 years	31
2	Gender	Male	48
		Female	10
3	Site	Lateral border of tongue	29
		Upper alveolus and gingiva	02
		Lower alveolus and gingiva	07
		Retromolar region	01
		Buccal mucosa	18
		Hard palate	01
4	Clinical staging(cTNM)	Stage 1-2	11
		Stage 3-4	47
5	Clinical Nodal metstasis(cN)	N0	26
		N1	22
		N2	07
		N3	03
6	Pathological Staging (pTNM)	Stage 1-2	12
		Stage 3-4	46
7	Pathological Nodal metstasis(pN)	N0	21
		N1	19
		N2	08
		N3	10
8	Grading	Well differentiated	13
		Moderately differentiated	36
		Poor differentiated	09
9	Worst Pattern of Invasion (WPOI-5)	Absent	36
		Present	22
10	Perineural Invasion(PNI)	Absent	43
		Present	15
11	Lymphocytic inflammation	Mild	03
		Moderate	48
		Dense	07
12	Overall survival endpoint	Survival	36
		Death	22

1

**Table 2:** Table Association of percentage stromal TILs and DOI with clinical variables and histopathological parameters.

Parameters		Percentage Stromal TILs				DOI			
		N	Mean±SD	SE	p-value	N	Mean±SD	SE	p-value
Age groups	≤30	3	11.14±2.74	1.58	0.001	3	1.10±0.20	0.11	0.152
	31-40	10	16.39±11.69	3.69		10	1.32±0.48	0.15	
	41-50	14	24.34±14.11	3.77		12	0.73±0.34	0.09	
	51-60	13	16.77±6.28	1.74		12	1.38±1.05	0.30	
	>60	18	31.18±11.82	2.78		18	1.13±0.58	0.13	
	Total	58	22.71±12.75	1.67		55	1.13±0.67	0.09	
Gender	Male	48	23.60±13.47	1.94	0.249	46	1.15±0.70	0.10	0.553
	Female	10	18.45±7.57	2.39		9	1.01±0.53	0.17	
Site	Lateral border of tongue	29	22.37±12.86	2.38	0.717	28	1.13±0.56	0.10	0.001
	Upper alveolus and gingiva	2	23.92±8.97	6.34		2	.075±0.35	0.25	
	Lower alveolus and gingiva	7	24.90±16.56	6.26		7	1.37±0.76	0.29	
	Retromolar region	1	7.88± 0	-		1	4.00±0.00	0.00	
	Buccal mucosa	18	22.27±11.93	2.81		16	0.89±0.39	0.09	
	Hard palate	1	37.69± 0	-		1	1.20±0.00	0.00	
	Total	58	22.71±12.75	1.67		55	1.13±0.67	0.09	
pStaging	Stage 1	2	12.47±10.42	7.36	0.571	2	1.05±0.49	0.35	0.044
	Stage 2	10	22.16±14.75	4.66		10	0.77±0.30	0.09	
	Stage 3	18	25.19±12.19	2.87		17	0.96±0.40	0.09	
	Stage 4	28	22.05±12.68	2.39		26	1.39±0.83	0.16	
	Total	58	22.71±12.75	1.67		55	1.13±0.67	0.09	
pN	N0	21	26.65±13.75	3.00	0.079	20	0.79±0.30	0.06	0.003
	N1	19	23.88±12.83	2.94		18	1.17±0.58	0.13	
	N2	8	13.92±5.58	1.97		8	1.78±1.13	0.40	
	N3	10	19.27±11.64	3.68		9	1.23±.50	0.16	
	Total	58	22.71±12.75	1.67		55	1.13±.67	0.09	
cStaging	Stage 1	1	19.84±-	-	0.622	1	0.70±0.00	0.00	0.154
	Stage 2	10	27.43±15.05	4.76		10	0.73±0.25	0.08	
	Stage 3	29	22.34±12.71	2.36		27	1.27±0.59	0.11	
	Stage 4	18	20.86±11.91	2.80		17	1.17±0.87	0.21	
	Total	58	22.71±12.75	1.67		55	1.13±0.67	0.09	
cN	N0	26	25.65±13.42	2.63	0.378	24	1.07±0.78	0.16	0.612
	N1	22	21.04±12.511	2.66		21	1.28±0.65	0.14	
	N2	7	17.02±10.417	3.93		7	0.97±0.40	0.15	
	N3	3	22.83±12.58	7.26		3	0.96±0.11	0.06	
	Total	58	22.71±12.75	1.67		55	1.13±0.67	0.09	
Grading	Well differentiated	13	26.28±13.80	3.82	0.498	13	0.89±0.39	0.11	0.256
	Moderately differentiated	36	21.35±12.25	2.04		34	1.24±0.79	0.13	
	Poorly differentiated	9	23.01±13.70	4.56		8	1.05±0.32	0.11	
	Total	58	22.71±12.75	1.674		55	1.13±0.67	0.09	
Modified BG Risk Score	Low Risk	-	-	-		37	0.98±0.59	0.09	0.020
	High Risk	-	-	-		18	1.43±0.74	0.17	

2

**Table 3:** Table Association of clinical variables and histopathological parameters with modified BG score and modified BG score risk categories.

Parameters		Modified BG score					p-value	Modified BG score risk categories			p-value
		0	1	2	3	Total		Low Risk	High Risk	Total	
Age groups	≤30	0	1	1	1	3	0.159	1	2	3	0.650
	31-40	2	4	1	3	10		6	4	10	
	41-50	4	6	4	0	14		10	4	14	
	51-60	4	6	0	3	13		10	3	13	
	>60	9	3	5	1	18		12	6	18	
	Total	19	20	11	8	58		39	19	58	
Gender	Male	15	15	10	8	48	0.360	30	18	48	0.092
	Female­­­­­­	4	5	1	0	10		9	1	10	
	Total	19	20	11	8	58		39	19	58	
Site	Lateral border of tongue	10	12	5	2	10	0.492	22	7	29	0.491
	Upper alveolus and gingiva	0	1	1	0	2		1	1	2	
	Lower alveolus and gingiva	3	1	1	2	7		4	3	7	
	Retromolar region	0	0	0	1	1		0	1	1	
	Buccal mucosa	5	6	4	3	18		11	7	18	
	Hard palate	1	0	0	0	1		1	0	1	
	Total	19	20	11	8	58		39	19	58	
p Staging	Stage 1	1	0	0	1	2	0.346	1	1	2	0.116
	Stage 2	5	4	1	0	10		9	1	10	
	Stage 3	7	7	3	1	18		14	4	18	
	Stage 4	6	9	7	6	28		15	13	28	
	Total	19	20	11	8	58		39	19	58	
pN	N0	9	9	2	1	9	0.005	18	3	21	0.022
	N1	7	6	4	2	19		13	6	19	
	N2	1	4	1	2	8		5	3	8	
	N3	2	1	4	3	10		3	7	10	
	Total	19	20	11	8	58		39	19	58	
cStaging	Stage 1	1	0	0	0	1	0.106	1	0	1	0.023
	Stage 2	7	3	0	0	10		10	0	10	
	Stage 3	8	10	5	6	29		18	11	29	
	Stage 4	3	7	6	2	18		10	8	18	
	Total	19	20	11	8	58		39	19	58	
cN	N0	12	9	2	3	26	0.188	21	5	26	0.018
	N1	5	9	4	4	5		14	8	22	
	N2	1	2	3	1	7		3	4	7	
	N3	1	0	2	0	3		1	2	3	
	Total	19	20	11	8	58		39	19	58	
Grading	Well differentiated	9	3	1	0	13	0.025	12	1	13	0.041
	Moderately differentiated	8	12	8	8	36		20	16	36	
	Poorly differentiated	2	5	2	0	9		7	2	9	
	Total	19	20	11	8	58		39	19	58	

3

## Data Availability

The datasets used and/or analyzed during the present study are available from the corresponding author.
